# Documenting fishes in an inland sea with citizen scientist diver surveys: using taxonomic expertise to inform the observation potential of fish species

**DOI:** 10.1007/s10661-022-09857-1

**Published:** 2022-02-26

**Authors:** Elizabeth A. Ashley, Christy V. Pattengill-Semmens, James W. Orr, Janna D. Nichols, Joseph K. Gaydos

**Affiliations:** 1 Karen C. Drayer Wildlife Health Center – Orcas Island Office, SeaDoc Society, 942 Deer Harbor Rd, Eastsound, WA 98245 USA; 2 Reef Environmental Education Foundation (REEF), Key Largo, PO Box 370246, FL, 33037 USA; 3 School of Aquatic and Fishery Sciences, University of Washington, Seattle, WA 98105 USA

**Keywords:** Citizen science, Fish distribution, Ichthyofauna, REEF Volunteer Fish Survey Project, Roving diver technique, Salish Sea

## Abstract

Long-term monitoring enables scientists and managers to track changes in the temporal and spatial distributions of fishes. Given the anthropogenic stressors affecting marine ecosystem health, there is a critical need for robust, comprehensive fish monitoring programs. Citizen science can serve as a meaningful, cost-effective strategy to survey fish communities. We compared data from 13,000 surveys collected over 21 years (1998–2019) by Reef Environmental Education Foundation (REEF) volunteer divers to a published compilation of Salish Sea ichthyofauna collected using an assortment of methods. Volunteer divers observed 138 of 261 recognized species in the Salish Sea, expanded the range of 18 species into additional Salish Sea sub-basins, and identified one species novel to the Salish Sea (*Gibbonsia metzi —* Striped Kelpfish). To identify Salish Sea fish species that are most suitable to be monitored by underwater visual census and to evaluate confidence in in situ identification, we developed a categorization system based on the likelihood of recreational divers and snorkelers encountering a given species, and on whether identification required a specimen in hand or could be classified to species visually (with or without a high-quality photograph). REEF divers encountered 62% (138 of 223) of the visually detectable species occurring in the region and 85% (102 of 120) of species most likely to be observed by recreational divers. Our findings show that citizen scientists provide valuable monitoring data for over half of the 261 marine and anadromous fish species known to occupy the Salish Sea, many of which are not routinely monitored otherwise.

**Supplementary information:**

The online version contains supplementary material available at 10.1007/s10661-022-09857-1.

## Introduction

Comprehensive monitoring efforts are integral to marine ecosystem management and to predicting and mitigating the effects of anthropogenic change on marine biodiversity. Traditionally, fish occupancy is determined using a combination of fishery-dependent (i.e., catch data) and fishery-independent (e.g., remote underwater video, satellite imagery, hydroacoustics, visual transects, point-count surveys, roving diver surveys) monitoring (Murphy & Jenkins, 2010). The efficacy of a given monitoring technique depends on the target species and its unique characteristics, including size, behavior, crypticity, swimming speed, preferred habitat, and position in the water column (Holt et al., 2013; Harmelin-Vivien & Francour, 1992). Additionally, as observational methods vary in cost, invasiveness, and ease, choosing the appropriate monitoring tool depends on budgets and specific objectives of managers or researchers. It is useful to assess the benefits and disadvantages of different techniques to identify combinations that can maximize surveillance of target species, communities, or habitats while adhering to financial and other constraints.

Underwater visual census (UVC) is a minimally invasive, fishery-independent method in which snorkel or SCUBA is used to assess biodiversity and/or biomass in marine habitats (Pattengill-Semmens & Semmens, 2003). The roving diver technique (RDT; Schmitt & Sullivan, 1996) is a type of UVC especially suited for detecting shy, cryptic, or demersal fishes difficult to assess with other monitoring tools (Murphy & Jenkins, 2010). During RDT surveys, divers swim freely throughout a dive site while recording each observed fish species and a corresponding log-scale categorical abundance. The method can be conducted in a variety of habitats in depths up to 30 m (and beyond with specialized gear). As such, RDT is valuable for assessing frequencies of occurrence, spatial distributions, abundance estimates, and information on status and trends for a broad array of species. Data collected with other visual methods, such as belt transects, can be combined with RDT data to provide a holistic picture that includes species densities and size distributions (Holt et al., 2013).

Across the globe, citizen science programs train recreational divers to effectively monitor marine fishes, invertebrates, and algae (Branchini et al., 2015; Cerrano et al., 2016). The Reef Environmental Education Foundation (REEF) Volunteer Fish Survey Project trains volunteer divers and snorkelers to independently conduct RDT surveys, creating a system by which thousands of standardized surveys are conducted annually and recorded in a publicly accessible database (Pattengill-Semmens & Semmens, 2003). Within such a large pool of trained volunteers lies the potential for scientists and managers to increase their census capabilities by alleviating constraints on equipment, personnel, time, and accessibility to certain areas or habitats. Since 1993, the REEF Volunteer Fish Survey Project has been used worldwide to assess biodiversity and advance the conservation of threatened fishes and invertebrates, both alone and in combination with professional research efforts (Holt et al., 2013; Thorson et al., 2014). Data collected by REEF surveyors have informed numerous conservation and management efforts, including to detect and remove invasive species (Smith et al., 2017), discover new species (Allen et al., 2020), contribute to the recovery of threatened species (Tolimieri et al., 2017), document the impact of human population density on fish communities (Stallings, 2009), and trace the impacts of enigmatic marine diseases (Harvell et al., 2019).

To determine if RDT is an appropriate monitoring tool for a fish species, multiple factors surrounding the species of interest must be considered. This includes, but is not limited to, whether the species occurs at recreational diving depths, is cryptic or conspicuous, and if it can be discerned from closely related species without a specimen in hand (e.g., for meristic counts). Identifying the species that volunteers can potentially encounter and visually identify can improve the interpretation of data provided by this no-cost, high-value monitoring tool (Schmitt & Sullivan, 1996).

The Salish Sea is a 16,925 km^2^ inland sea bordered by Washington, USA, and British Columbia, Canada. In 2019, relying on preserved specimens in archival institutions, published records, and, especially for within-basin distributions, unpublished field notes and logbooks, Pietsch and Orr (2019) published species accounts for 260 fish species found in the Salish Sea, including distributions across sub-basins. As many of these taxonomic records were generated over a century ago, this compilation focused on providing a comprehensive, cumulative registry of Salish Sea ichthyofauna in addition to a current picture of biodiversity. Records were based on collections of specimens using an array of gear and techniques, including beach and purse seines, trawls, gillnets, traps, spearfishing, hook and lines, poisoning, stomach contents, and beach casts (e.g., Miller & Borton, 1980). Data from RDT or other visual surveys were not included. This compilation provides the best available baseline of the total fish that use, or have used, the Salish Sea ecosystem. While no one method will be suitable to capture all species, individual techniques such as RDT can be evaluated against this list to elucidate the scope of the survey tool.

Since 1998, REEF divers have conducted RDT surveys in the Salish Sea. We compiled REEF RDT surveys recorded over a span of 21 years (1998–2019) and compared these data to the Pietsch and Orr (2019) records of 260 Salish Sea fish species. Because relatively few specimens are collected and preserved from rocky reefs and rock walls (Pietsch & Orr, 2019), often a focus area for recreational divers, we hypothesized that observations by REEF surveyors would expand the geographic range and number of species documented in the Salish Sea based on Pietsch and Orr (2019). Certain fish species are inherently better suited than others for identification by RDT. Therefore, to identify Salish Sea fish species that can be monitored by REEF divers, we developed a categorization system based on the potential for recreational divers and snorkelers to encounter them, and on whether they can be visually identified to species with or without a high-quality photograph if encountered (i.e., a specimen in hand is not required for identification).

## Methods

### REEF Volunteer Fish Survey Project

We compiled REEF RDT survey data collected by volunteer divers in the Salish Sea from 1998 to 2019 (REEF, 2020). REEF divers record fish species sightings on preprinted data sheets and enter the data online (or, prior to 2007, mailed in optical scan forms). General data for each survey includes surveyor ID, surveyor experience level (expert or novice), geographic location, survey date, and a variety of environmental variables (e.g., habitat, depth, and visibility). Fish species sighted are recorded on a checklist, and for each, abundance is noted by one of four log-scale abundance index categories: “Single” (1 individual), “Few” (2–10 individuals), “Many” (11–100 individuals), or “Abundant” (> 100 individuals). After performing quality control (Pattengill-Semmens & Semmens, 2003), REEF staff transfer the data into a publicly accessible database (www.REEF.org). REEF categorizes surveyors as expert or novice based on their experience level and performance on a series of fish identification examinations. For this study, we included only surveys collected by expert surveyors. We excluded sightings of fishes identified to genus but not to species.

### REEF vs. Pietsch and Orr comparison

The coordinates for each REEF survey location were used to group surveys into one of six oceanographic sub-basins of the Salish Sea: Central Basin (CB), Hood Canal (HC), Northern Straits (NS), South Puget Sound (SPS), Strait of Georgia (SOG), or Whidbey Basin (WB) (Moore et al., 2008; Thomson, 1994; Supplementary Map). We adapted the eight regions identified by Pietsch and Orr (2019) to these six sub-basins. A sub-basin was considered occupied by a given species if at least one expert-level REEF surveyor recorded the species there. Our list of REEF records in Table 1 is therefore a record of presence at any point in time between 1998 and 2019.

**Table 1:** Orders, families, and common and scientific names of fish species recorded in sub-basins of the Salish Sea, Strait of Georgia (SOG), Northern Straits (NS), Whidbey Basin (WB), Central Basin (CB), Hood Canal (HC), and South Puget Sound (SPS), by Reef Environmental Education Foundation (REEF) expert-level volunteer divers and by Pietsch and Orr (2019). Pietsch and Orr is designated by “PO.” An “X” indicates presence in a sub-basin; “#” indicates presence where REEF sightings expanded the known range of a species to another sub-basin within the Salish Sea. The identification category of each species describing its detectability in situ, as well as its sub-category (a–c) as per the identification key used in this study, is indicated by “ID”

Order	Family	Scientific name	Common name	CB		HC		NS		SOG		SPS		WB		ID
				REEF	PO	REEF	PO	REEF	PO	REEF	PO	REEF	PO	REEF	PO	
Acipenseriformes	Acipenseridae	*Acipenser medirostris*	Green Sturgeon						X		X					2a
Acipenseriformes	Acipenseridae	*Acipenser transmontanus*	White Sturgeon	X	X		X		X		X		X		X	1a
Anguilliformes	Nemichthyidae	*Nemichthys scolopaceus*	Slender Snipe Eel		X				X		X				X	2a
Argentiniformes	Bathylagidae	*Leuroglossus schmidti*	Northern Smoothtongue								X					2a
Atheriniformes	Atherinopsidae	*Atherinops affinis*	Topsmelt						X							1a
Aulopiformes	Alepisauridae	*Alepisaurus ferox*	Longnose Lancetfish		X				X		X		X		X	2a
Aulopiformes	Paralepididae	*Arctozenus risso*	White Barracudina		X								X		X	2a
Aulopiformes	Synodontidae	*Synodus lucioceps*	California Lizardfish		X				X				X		X	2a
Batrachoidiformes	Batrachoididae	*Porichthys notatus*	Plainfin Midshipman	X	X	X	X	X	X	X	X	X	X	X	X	1a
Beloniformes	Scomberesocidae	*Cololabis saira*	Pacific Saury		X				X						X	2a
Carcharhiniformes	Carcharhinidae	*Prionace glauca*	Blue Shark		X										X	1a
Carcharhiniformes	Scyliorhinidae	*Apristurus brunneus*	Brown Cat Shark		X		X		X		X		X		X	2a
Carcharhiniformes	Triakidae	*Triakis semifasciata*	Leopard Shark						X							1a
Chimaeriformes	Chimaeridae	*Hydrolagus colliei*	Spotted Ratfish	X	X	X	X	X	X	X	X	X	X	X	X	1a
Clupeiformes	Clupeidae	*Alosa sapidissima*	American Shad		X		X		X		X		X		X	2b
Clupeiformes	Clupeidae	*Clupea pallasii*	Pacific Herring	X	X	X	X	X	X	X	X	X	X	X	X	1a
Clupeiformes	Clupeidae	*Sardinops sagax*	Pacific Sardine		X		X	X	X	X	X		X		X	2a
Clupeiformes	Engraulidae	*Engraulis mordax*	Northern Anchovy	X	X	X	X	X	X	X	X	X	X		X	1a
Cottiformes	Pholidae	*Apodichthys flavidus*	Penpoint Gunnel	X	X	X	X	X	X	X	X	X	X	X	X	2c
Cottiformes	Agonidae	*Agonopsis vulsa*	Northern Spearnose Poacher	X	X	X	X	X	X	X	X	X	X	X	X	1a
Cottiformes	Agonidae	*Anoplagonus inermis*	Smooth Alligatorfish	X	X			X	X	X	X			X	X	1a
Cottiformes	Agonidae	*Bathyagonus alascanus*	Gray Starsnout		X				X		X		X		X	2b
Cottiformes	Agonidae	*Bathyagonus infraspinatus*	Spinycheek Starsnout		X		X		X		X				X	2b
Cottiformes	Agonidae	*Bathyagonus nigripinnis*	Blackfin Poacher		X		X		X		X				X	2b
Cottiformes	Agonidae	*Bathyagonus pentacanthus*	Bigeye Poacher	X	X				X		X		X	X	X	2b
Cottiformes	Agonidae	*Bothragonus swanii*	Rockhead		X			X	X						X	1a
Cottiformes	Agonidae	*Chesnonia verrucosa*	Warty Poacher		X				X						X	1b
Cottiformes	Agonidae	*Hypsagonus mozinoi*	Kelp Poacher						X							1b
Cottiformes	Agonidae	*Hypsagonus quadricornis*	Fourhorn Poacher		X				X						X	1a
Cottiformes	Agonidae	*Odontopyxis trispinosa*	Pygmy Poacher	X	X	X	X	X	X	X	X	X	X	X	X	1b
Cottiformes	Agonidae	*Pallasina aix*^*a*^	Southern Tubenose Poacher		X			X	X	X	X				X	1b
Cottiformes	Agonidae	*Podothecus accipenserinus*	Sturgeon Poacher	X	X	X	X	X	X	X	X	X	X	X	X	1b
Cottiformes	Agonidae	*Stellerina xyosterna*	Pricklebreast Poacher						X							3a
Cottiformes	Agonidae	*Xeneretmus latifrons*	Blacktip Poacher	X	X	X	X		X		X		X	X	X	1b
Cottiformes	Agonidae	*Xeneretmus leiops*	Smootheye Poacher						X							3a
Cottiformes	Agonidae	*Xeneretmus triacanthus*	Bluespotted Poacher		X		X		X				X		X	2b
Cottiformes	Anarhichadidae	*Anarrhichthys ocellatus*	Wolf-eel	X	X	X	X	X	X	X	X	X	X	X	X	1a
Cottiformes	Anoplopomatidae	*Anoplopoma fimbria*	Sablefish		X		X		X		X		X		X	2a
Cottiformes	Bathymasteridae	*Ronquilus jordani*	Northern Ronquil	X	X	X	X	X	X	X	X	X	X	X	X	1a
Cottiformes	Cottidae	*Artedius fenestralis*	Padded Sculpin	X	X	X	X	X	X	X	X	X	X	X	X	2c
Cottiformes	Cottidae	*Artedius harringtoni*	Scalyhead Sculpin	X	X	X	X	X	X	X	X	X	X	X	X	1b
Cottiformes	Cottidae	*Artedius lateralis*	Smoothhead Sculpin	X	X	X	X	X	X	X	X	X	X	X	X	1b
Cottiformes	Cottidae	*Artedius notospilotus*	Bonehead Sculpin		X										X	3a
Cottiformes	Cottidae	*Ascelichthys rhodorus*	Rosylip Sculpin		X			X	X	X	X		X		X	1a
Cottiformes	Cottidae	*Asemichthys taylori*	Spinynose Sculpin	#		#		X	X	X	X	#		#		2c
Cottiformes	Cottidae	*Chitonotus pugetensis*	Roughback Sculpin	X	X	X	X	X	X	X	X	X	X	X	X	1a
Cottiformes	Cottidae	*Clinocottus acuticeps*	Sharpnose Sculpin		X		X	X	X	X	X	X	X		X	2c
Cottiformes	Cottidae	*Clinocottus embryum*	Calico Sculpin		X				X		X		X		X	2b
Cottiformes	Cottidae	*Clinocottus globiceps*	Mosshead Sculpin		X				X						X	2b
Cottiformes	Cottidae	*Cottus aleuticus*	Coastrange Sculpin		X		X		X		X		X		X	2b
Cottiformes	Cottidae	*Cottus asper*	Prickly Sculpin		X		X		X		X		X		X	2b
Cottiformes	Cottidae	*Enophrys bison*	Buffalo Sculpin	X	X	X	X	X	X	X	X	X	X	X	X	1a
Cottiformes	Cottidae	*Hemilepidotus hemilepidotus*	Red Irish Lord	X	X	X	X	X	X	X	X	X	X	X	X	1a
Cottiformes	Cottidae	*Hemilepidotus spinosus*	Brown Irish Lord	X	X			X	X	#					X	2b
Cottiformes	Cottidae	*Icelinus borealis*	Northern Sculpin	X	X	X	X	X	X	X	X	X	X		X	1b
Cottiformes	Cottidae	*Icelinus burchami*	Dusky Sculpin				X		X		X					3a
Cottiformes	Cottidae	*Icelinus filamentosus*	Threadfin Sculpin		X	X	X		X	X	X		X		X	1b
Cottiformes	Cottidae	*Icelinus fimbriatus*	Fringed Sculpin								X					3b
Cottiformes	Cottidae	*Icelinus tenuis*	Spotfin Sculpin	X	X	X	X	#		X	X		X	X	X	1b
Cottiformes	Cottidae	*Jordania zonope*	Longfin Sculpin	X	X	#		X	X	X	X	X	X	X	X	1a
Cottiformes	Cottidae	*Leptocottus armatus*	Pacific Staghorn Sculpin	X	X	X	X	X	X	X	X	X	X	X	X	1a
Cottiformes	Cottidae	*Myoxocephalus polyacanthocephalus*	Great Sculpin	X	X	X	X	X	X	X	X	X	X	X	X	1a
Cottiformes	Cottidae	*Oligocottus maculosus*	Tidepool Sculpin	X	X	X	X	X	X	X	X	X	X	X	X	2c
Cottiformes	Cottidae	*Oligocottus rimensis*	Saddleback Sculpin		X				X		X				X	2c
Cottiformes	Cottidae	*Oligocottus snyderi*	Fluffy Sculpin						X	#						2c
Cottiformes	Cottidae	*Paricelinus hopliticus*	Thornback Sculpin							X	X					2b
Cottiformes	Cottidae	*Radulinus asprellus*	Slim Sculpin	X	X	X	X	X	X	X	X		X		X	2b
Cottiformes	Cottidae	*Radulinus boleoides*	Darter Sculpin		X				X				X			2b
Cottiformes	Cottidae	*Ruscarius meanyi*	Puget Sound Sculpin		X				X	X	X				X	2b
Cottiformes	Cottidae	*Scorpaenichthys marmoratus*	Cabezon	X	X	X	X	X	X	X	X	X	X	X	X	1a
Cottiformes	Cottidae	*Synchirus gilli*	Manacled Sculpin	X	X		X	X	X	X	X	X	X		X	2b
Cottiformes	Cottidae	*Triglops macellus*	Roughspine Sculpin	X	X	X	X	X	X	X	X		X		X	1b
Cottiformes	Cottidae	*Triglops pingelii*	Ribbed Sculpin	X	X			X	X	X	X				X	1b
Cottiformes	Cryptacanthodidae	*Cryptacanthodes aleutensis*	Dwarf Wrymouth		X		X		X		X		X		X	3b
Cottiformes	Cryptacanthodidae	*Cryptacanthodes giganteus*	Giant Wrymouth		X				X		X				X	1a
Cottiformes	Cyclopteridae	*Eumicrotremus orbis*	Pacific Spiny Lumpsucker	X	X			X	X	X	X	X	X		X	1a
Cottiformes	Hemitripteridae	*Blepsias cirrhosus*	Silverspotted Sea Raven	X	X		X	X	X	X	X	X	X		X	1a
Cottiformes	Hemitripteridae	*Nautichthys oculofasciatus*	Sailfin Sea Raven	X	X	X	X	X	X	X	X	X	X	X	X	1a
Cottiformes	Hexagrammidae	*Hexagrammos decagrammus*	Kelp Greenling	X	X	#		X	X	X	X	X	X	X	X	1a
Cottiformes	Hexagrammidae	*Hexagrammos lagocephalus*	Rock Greenling	X	X	#		X	X	X	X		X		X	2b
Cottiformes	Hexagrammidae	*Hexagrammos stelleri*	Whitespotted Greenling	X	X	X	X	X	X	X	X	X	X	X	X	1a
Cottiformes	Hexagrammidae	*Ophiodon elongatus*	Lingcod	X	X	X	X	X	X	X	X	X	X	X	X	1a
Cottiformes	Hexagrammidae	*Oxylebius pictus*	Painted Greenling	X	X	X	X	X	X	X	X	X	X	X	X	1a
Cottiformes	Hexagrammidae	*Zaniolepis latipinnis*	Longspine Combfish	X	X	X	X		X	X	X	X	X	X	X	1a
Cottiformes	Liparidae	*Careproctus melanurus*	Blacktail Snailfish		X				X		X				X	3a
Cottiformes	Liparidae	*Liparis callyodon*	Spotted Snailfish	X	X			X	X		X				X	2c
Cottiformes	Liparidae	*Liparis cyclopus*	Ribbon Snailfish		X				X		X		X		X	2c
Cottiformes	Liparidae	*Liparis dennyi*	Marbled Snailfish	X	X			X	X		X		X	X	X	2c
Cottiformes	Liparidae	*Liparis florae*	Tidepool Snailfish	X	X			X	X		X				X	2c
Cottiformes	Liparidae	*Liparis fucensis*	Slipskin Snailfish	X	X				X		X		X		X	2c
Cottiformes	Liparidae	*Liparis greeni*	Lobefin Snailfish	#				X	X		X	#		#		2c
Cottiformes	Liparidae	*Liparis mucosus*	Slimy Snailfish						X							3a
Cottiformes	Liparidae	*Liparis pulchellus*	Showy Snailfish	X	X		X	X	X	X	X		X	X	X	1a
Cottiformes	Liparidae	*Liparis rutteri*	Ringtail Snailfish						X		X					3a
Cottiformes	Liparidae	*Lipariscus nanus*	Pygmy Snailfish								X					3a
Cottiformes	Liparidae	*Nectoliparis pelagicus*	Tadpole Snailfish		X		X				X		X		X	3a
Cottiformes	Liparidae	*Paraliparis deani*	Prickly Snailfish								X					3a
Cottiformes	Pholidae	*Apodichthys fucorum*	Rockweed Gunnel	X	X			X	X	X	X		X		X	2b
Cottiformes	Pholidae	*Pholis clemensi*	Longfin Gunnel	X	X	X	X	X	X	X	X	#		X	X	1a
Cottiformes	Pholidae	*Pholis laeta*	Crescent Gunnel	X	X	X	X	X	X	X	X	X	X	X	X	2c
Cottiformes	Pholidae	*Pholis ornata*	Saddleback Gunnel	X	X	X	X	X	X	X	X	X	X	X	X	2c
Cottiformes	Pholidae	*Pholis schultzi*	Red Gunnel	X	X			X	X			#			X	2b
Cottiformes	Psychrolutidae	*Dasycottus setiger*	Spiny Fathead	X	X		X	X	X	X	X		X	X	X	1a
Cottiformes	Psychrolutidae	*Malacocottus kincaidi*	Blackfin Fathead				X				X					3b
Cottiformes	Psychrolutidae	*Malacocottus zonurus*	Darkfin Fathead						X							3b
Cottiformes	Psychrolutidae	*Psychrolutes paradoxus*	Tadpole Fathead	X	X		X	X	X	X	X		X		X	1a
Cottiformes	Psychrolutidae	*Psychrolutes sigalutes*	Soft Fathead	X	X	X	X	X	X	X	X		X		X	2b
Cottiformes	Ptilichthyidae	*Ptilichthys goodei*	Quillfish	X	X	X	X		X	X	X				X	1a
Cottiformes	Rhamphocottidae	*Rhamphocottus richardsonii*	Grunt Sculpin	X	X	X	X	X	X	X	X	X	X	X	X	1a
Cottiformes	Scytalinidae	*Scytalina cerdale*	Graveldiver						X							3a
Cottiformes	Stichaeidae	*Anoplarchus insignis*	Slender Cockscomb	X	X	#		X	X	X	X	X	X	X	X	2c
Cottiformes	Stichaeidae	*Anoplarchus purpurescens*	High Cockscomb	X	X	X	X	X	X	X	X	X	X	X	X	2c
Cottiformes	Stichaeidae	*Chirolophis decoratus*	Decorated Warbonnet	X	X	#		X	X	X	X	X	X	X	X	1a
Cottiformes	Stichaeidae	*Chirolophis nugator*	Mosshead Warbonnet	X	X	#		X	X	X	X	X	X	X	X	1a
Cottiformes	Stichaeidae	*Leptoclinus maculatus*	Daubed Shanny		X				X		X				X	1a
Cottiformes	Stichaeidae	*Lumpenella longirostris*	Longsnout Prickleback								X					2b
Cottiformes	Stichaeidae	*Lumpenopsis hypochroma*	Y-Prickleback								X					3a
Cottiformes	Stichaeidae	*Lumpenus sagitta*	Snake Prickleback	X	X	X	X	X	X	X	X	X	X	X	X	1a
Cottiformes	Stichaeidae	*Phytichthys chirus*	Ribbon Prickleback		X				X	X	X		X	X	X	2c
Cottiformes	Stichaeidae	*Plectobranchus evides*	Bluebarred Prickleback		X	X	X				X		X		X	1a
Cottiformes	Stichaeidae	*Poroclinus rothrocki*	Whitebarred Prickleback		X		X		X		X		X		X	2b
Cottiformes	Stichaeidae	*Xiphister atropurpureus*	Black Prickleback		X			X	X		X		X		X	2c
Cottiformes	Stichaeidae	*Xiphister mucosus*	Rock Prickleback	X	X			X	X	X	X				X	2c
Cottiformes	Zaproridae	*Zaprora silenus*	Prowfish		X				X		X		X			2a
Cottiformes	Zoarcidae	*Lycodapus mandibularis*	Pallid Eelpout		X		X				X		X		X	3a
Cottiformes	Zoarcidae	*Lycodapus parviceps*	Smallhead Eelpout						X		X					3a
Cottiformes	Zoarcidae	*Lycodes beringi*	Bering Eelpout		X		X		X		X				X	3b
Cottiformes	Zoarcidae	*Lycodes brevipes*	Shortfin Eelpout		X				X		X		X		X	2b
Cottiformes	Zoarcidae	*Lycodes cortezianus*	Bigfin Eelpout		X				X						X	3b
Cottiformes	Zoarcidae	*Lycodes pacificus*	Blackbelly Eelpout	X	X	X	X	X	X	X	X	X	X	X	X	2c
Cottiformes	Zoarcidae	*Lycodes palearis*	Wattled Eelpout		X		X		X		X				X	2c
Cypriniformes	Cyprinidae	*Cyprinus carpio*	Common Carp								X					2a
Cyprinodontiformes	Cyprinodontidae	*Cyprinodon variegatus*	Sheepshead Minnow						X							2a
Gadiformes	Gadidae	*Gadus macrocephalus*	Pacific Cod	X	X	X	X	X	X	X	X		X	X	X	1b
Gadiformes	Gadidae	*Microgadus proximus*	Pacific Tomcod	X	X	X	X	X	X	X	X	X	X	X	X	1b
Gadiformes	Gadidae	*Theragra chalcogramma*	Walleye Pollock	X	X	X	X	X	X	X	X	X	X	X	X	1b
Gadiformes	Merlucciidae	*Merluccius productus*	Pacific Hake		X	X	X		X		X		X		X	1a
Gasterosteiformes	Aulorhynchidae	*Aulorhynchus flavidus*	Tubesnout	X	X	X	X	X	X	X	X	X	X	X	X	1a
Gasterosteiformes	Gasterosteidae	*Gasterosteus aculeatus*	Threespine Stickleback	X	X	X	X	X	X	X	X		X	X	X	1a
Gasterosteiformes	Syngnathidae	*Syngnathus leptorhynchus*	Bay Pipefish	X	X	X	X	X	X	X	X	X	X	X	X	1a
Gobiesociformes	Gobiesocidae	*Gobiesox maeandricus*	Northern Clingfish	X	X	#		X	X	X	X	#			X	1a
Hexanchiformes	Hexanchidae	*Hexanchus griseus*	Bluntnose Sixgill Shark	X	X	X	X	X	X	X	X	X	X	X	X	1a
Hexanchiformes	Hexanchidae	*Notorynchus cepedianus*	Broadnose Sevengill Shark		X						X		X			2a
Lamniformes	Alopiidae	*Alopias vulpinus*	Common Thresher Shark						X							1a
Lamniformes	Cetorhinidae	*Cetorhinus maximus*	Basking Shark		X				X		X		X		X	1a
Lamniformes	Lamnidae	*Lamna ditropis*	Salmon Shark		X				X		X				X	1a
Lampriformes	Lampridae	*Lampris guttatus*	Opah		X				X						X	2a
Lampriformes	Trachipteridae	*Trachipterus altivelis*	King-of-the-Salmon		X		X		X		X		X			2a
Myctophiformes	Myctophidae	*Diaphus theta*	California Headlightfish		X		X		X		X		X		X	3a
Myctophiformes	Myctophidae	*Nannobrachium regale*	Pinpoint Lampfish								X					3a
Myctophiformes	Myctophidae	*Protomyctophum crockeri*	California Flashlightfish								X					3a
Myctophiformes	Myctophidae	*Protomyctophum thompsoni*	Northern Flashlightfish						X							3a
Myctophiformes	Myctophidae	*Stenobrachius leucopsarus*	Northern Lampfish		X				X				X		X	3a
Myctophiformes	Myctophidae	*Tarletonbeania crenularis*	Blue Lanternfish						X							3a
Myxiniformes	Myxinidae	*Eptatretus stoutii*	Pacific Hagfish		X				X						X	2a
Ophidiiformes	Bythitidae	*Brosmophycis marginata*	Red Brotula	X	X	X	X	X	X	X	X		X	X	X	1a
Ophidiiformes	Ophidiidea	*Chilara taylori*	Spotted Cusk-eel								X					1a
Osmeriformes	Osmeridae	*Allosmerus elongatus*	Whitebait Smelt						X							3a
Osmeriformes	Osmeridae	*Hypomesus pretiosus*	Surf Smelt	X	X	X	X	X	X	X	X		X		X	2b
Osmeriformes	Osmeridae	*Mallotus villosus*	Pacific Capelin						X							2b
Osmeriformes	Osmeridae	*Spirinchus starksi*	Night Smelt						X							3a
Osmeriformes	Osmeridae	*Spirinchus thaleichthys*	Longfin Smelt		X		X		X		X		X		X	2b
Osmeriformes	Osmeridae	*Thaleichthys pacificus*	Eulachon		X				X		X		X		X	2b
Perciformes	Ammodytidae	*Ammodytes personatus*	Pacific Sand Lance	X	X	X	X	X	X	X	X	X	X	X	X	1a
Perciformes	Bramidae	*Brama japonica*	Pacific Pomfret		X										X	2a
Perciformes	Carangidae	*Trachurus symmetricus*	Jack Mackerel		X								X			2a
Perciformes	Centrolophidae	*Icichthys lockingtoni*	Medusafish						X				X			2a
Perciformes	Clinidae	*Gibbonsia metzi*	Striped Kelpfish					#								1a
Perciformes	Embiotocidae	*Amphistichus rhodoterus*	Redtail Surfperch						X							3b
Perciformes	Embiotocidae	*Brachyistius frenatus*	Kelp Perch	X	X	X	X	X	X	X	X	X	X	X	X	1a
Perciformes	Embiotocidae	*Cymatogaster aggregata*	Shiner Perch	X	X	X	X	X	X	X	X	X	X	X	X	1a
Perciformes	Embiotocidae	*Embiotoca lateralis*	Striped Seaperch	X	X	X	X	X	X	X	X	X	X	X	X	1a
Perciformes	Embiotocidae	*Hyperprosopon ellipticum*	Silver Surfperch		X				X						X	2b
Perciformes	Embiotocidae	*Phanerodon furcatus*	White Seaperch		X		X		X		X				X	2b
Perciformes	Embiotocidae	*Rhacochilus vacca*	Pile Perch	X	X	X	X	X	X	X	X	X	X	X	X	1a
Perciformes	Gobiidae	*Clevelandia ios*	Arrow Goby		X		X		X		X		X		X	2b
Perciformes	Gobiidae	*Lepidogobius lepidus*	Bay Goby	X	X	X	X	X	X	X	X		X	X	X	2c
Perciformes	Gobiidae	*Rhinogobiops nicholsii*	Blackeye Goby	X	X	X	X	X	X	X	X	X	X	X	X	1a
Perciformes	Icosteidae	*Icosteus aenigmaticus*	Ragfish		X				X				X		X	2a
Perciformes	Moronidae	*Morone saxatilis*	Striped Bass		X				X				X		X	2a
Perciformes	Sciaenidae	*Atractoscion nobilis*	White Seabass		X				X		X				X	2b
Perciformes	Sciaenidae	*Genyonemus lineatus*	White Croaker		X						X		X		X	2b
Perciformes	Sciaenidae	*Seriphus politus*	Queenfish		X				X		X				X	2b
Perciformes	Scombridae	*Sarda chiliensis*	Pacific Bonito		X		X		X		X		X		X	3b
Perciformes	Scombridae	*Scomber japonicus*	Pacific Chub Mackerel		X				X		X		X		X	3b
Perciformes	Sphyraenidae	*Sphyraena argentea*	Pacific Barracuda		X				X				X		X	2a
Perciformes	Stromateidae	*Peprilus simillimus*	Pacific Pompano		X		X		X		X		X		X	1a
Perciformes	Trichiuridae	*Benthodesmus pacificus*	North Pacific Frostfish						X							3a
Perciformes	Trichiuridae	*Benthodesmus tenuis*	Slender Frostfish		X				X						X	3a
Perciformes	Trichodontidae	*Trichodon trichodon*	Pacific Sandfish		X				X		X				X	1a
Petromyzontiformes	Petromyzontidae	*Entosphenus tridentatus*	Pacific Lamprey		X		X		X		X		X		X	2b
Petromyzontiformes	Petromyzontidae	*Lampetra ayresii*	Western River Lamprey		X				X		X		X		X	2b
Pleuronectiformes	Cynoglossidae	*Symphurus atricaudus*	California Tonguefish						X							2b
Pleuronectiformes	Paralichthyidae	*Citharichthys sordidus*	Pacific Sanddab	X	X	X	X	X	X	X	X	X	X	X	X	1b
Pleuronectiformes	Paralichthyidae	*Citharichthys stigmaeus*	Speckled Sanddab	X	X	X	X	X	X	X	X	X	X	X	X	1b
Pleuronectiformes	Pleuronectidae	*Atheresthes stomias*	Arrowtooth Flounder		X		X		X		X		X		X	2b
Pleuronectiformes	Pleuronectidae	*Eopsetta jordani*	Petrale Sole		X		X		X		X		X		X	2b
Pleuronectiformes	Pleuronectidae	*Glyptocephalus zachirus*	Rex Sole	X	X	X	X		X		X		X		X	1b
Pleuronectiformes	Pleuronectidae	*Hippoglossoides elassodon*	Flathead Sole		X		X		X	X	X		X		X	1b
Pleuronectiformes	Pleuronectidae	*Hippoglossus stenolepis*	Pacific Halibut		X		X	X	X		X		X		X	1a
Pleuronectiformes	Pleuronectidae	*Isopsetta isolepis*	Butter Sole		X		X	X	X		X	X	X		X	2c
Pleuronectiformes	Pleuronectidae	*Lepidopsetta bilineata*	Southern Rock Sole	X	X	X	X	X	X	X	X	X	X	X	X	2c
Pleuronectiformes	Pleuronectidae	*Lepidopsetta polyxystra*	Northern Rock Sole		X		X		X		X		X		X	2c
Pleuronectiformes	Pleuronectidae	*Limanda aspera*	Yellowfin Sole						X							2c
Pleuronectiformes	Pleuronectidae	*Lyopsetta exilis*	Slender Sole	X	X	X	X	X	X	X	X	X	X	X	X	1b
Pleuronectiformes	Pleuronectidae	*Microstomus pacificus*	Dover Sole	X	X		X	X	X	X	X		X		X	1b
Pleuronectiformes	Pleuronectidae	*Parophrys vetulus*	English Sole	X	X	X	X	X	X	X	X	X	X	X	X	1b
Pleuronectiformes	Pleuronectidae	*Platichthys stellatus*	Starry Flounder	X	X	X	X	X	X	X	X	X	X	X	X	1b
Pleuronectiformes	Pleuronectidae	*Pleuronichthys coenosus*	C-O Sole	X	X	X	X	X	X	X	X	X	X	X	X	1a
Pleuronectiformes	Pleuronectidae	*Pleuronichthys decurrens*	Curlfin Sole		X				X				X		X	2b
Pleuronectiformes	Pleuronectidae	*Psettichthys melanostictus*	Sand Sole	X	X	X	X	X	X		X		X		X	1b
Rajiformes	Rajiformes	*Bathyraja interrupta*	Bering Skate								X					3a
Rajiformes	Rajiformes	*Bathyraja kincaidii*	Sandpaper Skate						X		X					3a
Rajiformes	Rajiformes	*Beringraja binoculata*	Big Skate	X	X	X	X	X	X	X	X	X	X	X	X	1a
Rajiformes	Rajiformes	*Raja inornata*	California Skate						X							1a
Rajiformes	Rajiformes	*Raja rhina*	Longnose Skate	X	X	X	X		X		X		X	X	X	1a
Salmoniformes	Salmonidae	*Oncorhynchus clarkii*	Cutthroat Trout	X	X		X		X		X		X		X	1a
Salmoniformes	Salmonidae	*Oncorhynchus gorbuscha*	Pink Salmon		X		X		X		X		X	X	X	1a
Salmoniformes	Salmonidae	*Oncorhynchus keta*	Chum Salmon		X		X		X	X	X		X		X	1a
Salmoniformes	Salmonidae	*Oncorhynchus kisutch*	Coho Salmon	X	X		X		X	X	X		X		X	1a
Salmoniformes	Salmonidae	*Oncorhynchus mykiss*	Steelhead (Rainbow Trout)		X		X		X		X		X	X	X	1a
Salmoniformes	Salmonidae	*Oncorhynchus nerka*	Sockeye Salmon		X		X		X		X		X		X	1a
Salmoniformes	Salmonidae	*Oncorhynchus tshawytscha*	Chinook Salmon	X	X		X		X		X		X	X	X	1a
Salmoniformes	Salmonidae	*Salmo salar*	Atlantic Salmon		X		X		X		X		X		X	1a
Salmoniformes	Salmonidae	*Salvelinus confluentus*	Bull Trout		X		X		X		X		X		X	2b
Salmoniformes	Salmonidae	*Salvelinus malma*	Dolly Varden		X		X		X		X		X		X	2b
Scorpaeniformes	Scorpaenidae	*Sebastes aleutianus*	Rougheye Rockfish		X				X						X	2a
Scorpaeniformes	Scorpaenidae	*Sebastes alutus*	Pacific Ocean Perch						X		X				X	2b
Scorpaeniformes	Scorpaenidae	*Sebastes auriculatus*	Brown Rockfish	X	X	X	X	X	X	X	X	X	X	X	X	1a
Scorpaeniformes	Scorpaenidae	*Sebastes babcocki*	Redbanded Rockfish				X		X							2a
Scorpaeniformes	Scorpaenidae	*Sebastes brevispinis*	Silvergray Rockfish	X	X	#		X	X				X			1a
Scorpaeniformes	Scorpaenidae	*Sebastes caurinus*	Copper Rockfish	X	X	X	X	X	X	X	X	X	X	X	X	1a
Scorpaeniformes	Scorpaenidae	*Sebastes crameri*	Darkblotched Rockfish		X				X		X				X	2a
Scorpaeniformes	Scorpaenidae	*Sebastes diaconus*	Deacon Rockfish			#		X	X	X	X					2c
Scorpaeniformes	Scorpaenidae	*Sebastes diploproa*	Splitnose Rockfish		X		X		X		X				X	2b
Scorpaeniformes	Scorpaenidae	*Sebastes elongatus*	Greenstriped Rockfish		X		X		X	X	X		X		X	1a
Scorpaeniformes	Scorpaenidae	*Sebastes emphaeus*	Puget Sound Rockfish	X	X	X	X	X	X	X	X	X	X	X	X	1a
Scorpaeniformes	Scorpaenidae	*Sebastes entomelas*	Widow Rockfish		X			X	X	#					X	1a
Scorpaeniformes	Scorpaenidae	*Sebastes flavidus*	Yellowtail Rockfish	X	X	X	X	X	X	X	X	X	X	X	X	1a
Scorpaeniformes	Scorpaenidae	*Sebastes helvomaculatus*	Rosethorn Rockfish		X				X						X	3b
Scorpaeniformes	Scorpaenidae	*Sebastes maliger*	Quillback Rockfish	X	X	X	X	X	X	X	X	X	X	X	X	1a
Scorpaeniformes	Scorpaenidae	*Sebastes melanops*	Black Rockfish	X	X	X	X	X	X	X	X	X	X	X	X	1a
Scorpaeniformes	Scorpaenidae	*Sebastes miniatus*	Vermilion Rockfish	X	X	X	X	X	X	X	X	X	X	X	X	1a
Scorpaeniformes	Scorpaenidae	*Sebastes nebulosus*	China Rockfish	X	X	#		X	X	X	X				X	1a
Scorpaeniformes	Scorpaenidae	*Sebastes nigrocinctus*	Tiger Rockfish	X	X			X	X	X	X				X	1a
Scorpaeniformes	Scorpaenidae	*Sebastes paucispinis*	Bocaccio	X	X	X	X	X	X	X	X		X		X	1a
Scorpaeniformes	Scorpaenidae	*Sebastes pinniger*	Canary Rockfish	X	X	X	X	X	X	X	X		X		X	1a
Scorpaeniformes	Scorpaenidae	*Sebastes proriger*	Redstripe Rockfish	X	X	X	X		X		X		X	X	X	1a
Scorpaeniformes	Scorpaenidae	*Sebastes rosaceus*	Rosy Rockfish		X				X						X	2b
Scorpaeniformes	Scorpaenidae	*Sebastes ruberrimus*	Yelloweye Rockfish	X	X	X	X	X	X	X	X		X	X	X	1a
Scorpaeniformes	Scorpaenidae	*Sebastes saxicola*	Stripetail Rockfish		X		X		X		X		X		X	3b
Scorpaeniformes	Scorpaenidae	*Sebastes semicinctus*	Halfbanded Rockfish						X							2a
Scorpaeniformes	Scorpaenidae	*Sebastes zacentrus*	Sharpchin Rockfish		X	X		X	X		X		X		X	2b
Scorpaeniformes	Scorpaenidae	*Sebastolobus alascanus*	Shortspine Thornyhead		X		X		X		X		X		X	2b
Squaliformes	Somniosidae	*Somniosus pacificus*	Pacific Sleeper Shark		X				X		X				X	2a
Squaliformes	Squalidae	*Squalus suckleyi*	Spotted Spiny Dogfish	X	X	X	X	X	X	X	X	X	X	X	X	1a
Squatiniformes	Squatinidae	*Squatina californica*	Pacific Angel Shark		X										X	1a
Stomiiformes	Sternoptychidae	*Argyropelecus sladeni*	Lowcrest Hatchetfish								X					3a
Stomiiformes	Stomiidae	*Chauliodus macouni*	Pacific Viperfish						X		X					2a
Tetraodontiformes	Molidae	*Mola mola*	Ocean Sunfish		X			X	X		X		X		X	1a
Torpediniformes	Torpedinidae	*Torpedo californica*	Pacific Electric Ray	X	X		X		X		X	X	X		X	1a

^*a*^Originally as *Pallasina barbata*, see Stevenson et al. (2021)

### Identification confidence categories

**Fig. 1:**
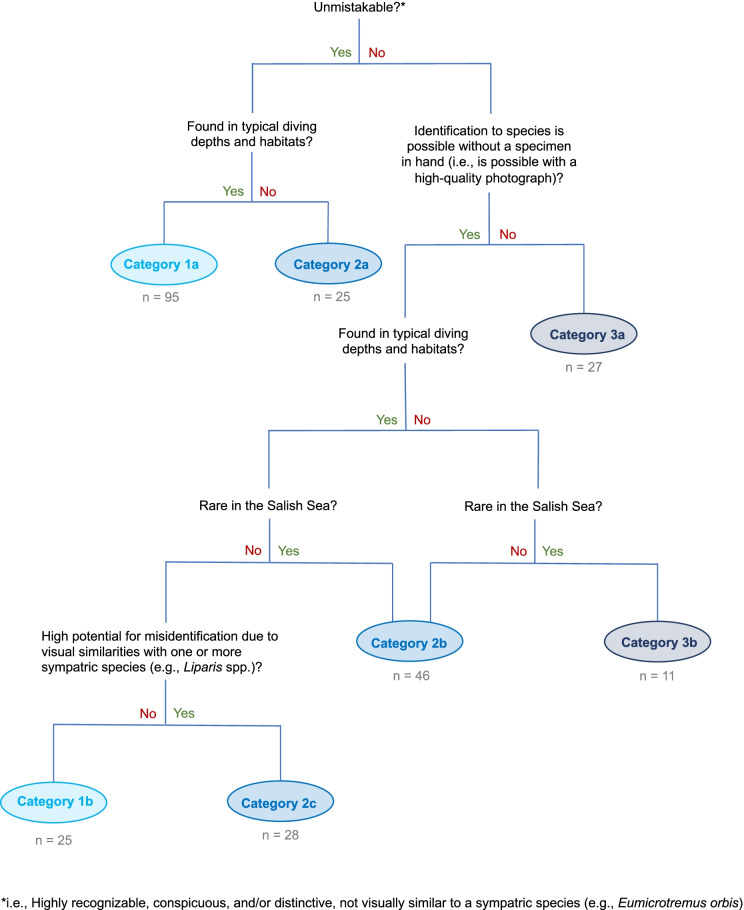
Identification key used to independently assign each Salish Sea fish species a detectability category for visual surveys. A given fish species can belong to one of three categories: category 1 — conspicuous and distinctive, with no photograph or specimen necessary for verification; category 2 — requires photographic evidence to be confidently reported on a visual survey; category 3 — has physical or life history characteristics that require a specimen in hand for identification to species. Sub-categories a, b, and c provide the rationale for assignment of a species to category 1, 2, or 3

We created an identification key to separate fishes known to inhabit the Salish Sea (Pietsch & Orr, 2019) into ranked categories indicating their suitability for visual census (Fig. 1) based on ease of visual identification and the potential for encounter on dives. For each species, we determined if a fish could be visually identified, and if so, whether the validity of a sighting benefits from a verifying photograph or if identification requires a specimen in hand. Species requiring a photograph included those that were rare or beyond diving range (not both), very similar in appearance to a sympatric species (e.g., *Icelinus borealis* — Northern Sculpin vs. *Icelinus fimbriatus* — Fringed Sculpin), or easily misidentified (*Liparis* spp. — Snailfishes). Fishes that required a specimen in hand for identification were those for which identification requires scale or spine counts or other measurements not possible from photographs (e.g., *Bathyagonus* spp. — Starsnouts).

As per the identification key (Fig. 1), category 1 fishes are unmistakable (category 1a) or are visually identifiable, occur at diving depths, and are not rare in the Salish Sea (category 1b). Category 2 fishes (a) are unmistakable but not typically found at diving depths; (b) are rare in the Salish Sea but live within diving depths (e.g., *Lumpenopsis hypochroma* — Y-Prickleback), or are not rare but exceed diving depths (e.g., *Lycodes palearis* — Wattled Eelpout); or (c) look very similar to other species (e.g., *Sarda chiliensis* — Pacific Bonito and *Scomber japonicus* — Pacific Chub Mackerel). These species would benefit from photographic evidence for confident reporting on a visual survey. Category 3 fish species have physical characteristics discernable only with a specimen in hand (category 3a) or are both rare in the Salish Sea and beyond diving depths (category 3b).

## Results

From 1998 to 2019, expert REEF divers conducted 13,274 Volunteer Fish Survey Project surveys in the Salish Sea (REEF, 2020). Most of these surveys were conducted in rocky habitats (52%), including rock or shale reefs and boulder fields, or on artificial reefs (35%), such as shipwrecks and other man-made structures. The remaining were performed over other habitat types including mud/silt, surfgrass and eelgrass, or open ocean. The largest percentage of surveys were recorded in Central Basin (39%), followed by Northern Straits (27%), the Strait of Georgia (15%), and Hood Canal (13%). South Puget Sound and Whidbey Basin comprised the smallest percentage of surveys (4% and 2%, respectively) (Supplemental Map).

Based on all REEF expert surveys conducted during the study period, REEF volunteers reported 137 of the 260 species listed in Pietsch and Orr (2019), as well as one additional species not included in the original species inventory. This brings the total number of fish species known to occur in the Salish Sea to 261. Table 1 lists the category and sub-category (e.g., 1a) of each species in accordance with the identification key (Fig. 1). We determined that 85% (*n* = 223) of the 261 species (Table 1) are visually identifiable (categories 1 and 2). Of the 223 species for which visual observation and subsequent positive identification is possible, 120 species are considered conspicuous and distinctive, not rare in the Salish Sea, and present in habitats likely to be visually surveyed by divers (category 1). These category 1 fishes can be reported with high confidence without a photograph and have the highest potential for documentation by REEF surveyors. The other 103 visually identifiable species were less likely to be documented on REEF surveys because they (1) are typically oceanic or found outside diving depths, (2) are considered vagrants or rare, (3) behave in a manner that evades visual detection by divers (e.g., cryptic, nocturnal), or (4) are difficult, but not impossible, to distinguish from sympatric species (category 2). In these cases, photographic evidence may be necessary to aid or confirm positive identification. The remaining 38 species known to occur in the Salish Sea (Pietsch & Orr, 2019) are assumed to require a specimen in hand for definitive identification or are extremely unlikely to be sighted due to rarity and bathypelagic or bathydemersal habitat preferences (category 3). From 1998 to 2019, REEF divers sighted 85% (102 of 120) of category 1 species, and 35% (36 of 103) of category 2 species.

We identified one novel species, *Gibbonsia metzi* — Striped Kelpfish, that had valid REEF sightings in the Salish Sea but was not included in Pietsch and Orr (2019). REEF divers documented this species near Cape Flattery, Washington, once in 2006 and again in 2007. This species is easily recognizable (category 1) and commonly sighted on the outer coasts of Vancouver Island and Washington, just beyond the Salish Sea’s westernmost border.

Consistent with our hypothesis, RDT surveys helped to fill knowledge gaps in the spatial distributions of species inhabiting rocky reef or kelp habitats at diving depths. REEF divers recorded the novel presence of ten category 1 species in one or more basins previously not documented by Pietsch and Orr (2019): *Sebastes brevispinis* — Silvergray Rockfish, *S. emphaeus* — Widow Rockfish, *S. flavidus* — China Rockfish; *Hexagrammos decagrammus* — Kelp Greenling, *Chirolophis decoratus* — Decorated Warbonnet, *C. nugator* — Mosshead Warbonnet, *Pholis clemensi* — Longfin Gunnel, *Gobiesox maeandricus* — Northern Clingfish, *Icelinus tenuis* — Spotfin Sculpin, and *Jordania zonope* — Longfin Sculpin. Similarly, REEF survey data showed that eight category 2 species were found to have a wider range within the Salish Sea: *Asemichthys taylori* — Spinynose Sculpin, *Hemilepidotus spinosus* — Brown Irish Lord, *Oligocottus snyderi* — Fluffy Sculpin, *Hexagrammos lagocephalus* — Rock Greenling, *Liparis greeni* — Lobefin Snailfish, *Sebastes diaconus* — Deacon Rockfish, *Anoplarchus insignis* — Slender Cockscomb, and *Pholis schultzi* — Red Gunnel. These 18 species are denoted with a “#” in Table 1.

## Discussion

In an era in which marine ecosystems are at risk from a suite of anthropogenic stressors, it is critical to strengthen biodiversity monitoring efforts that can inform policy and management (Friedman et al., 2020). Using robust citizen science to document species distributions can permit more comprehensive tracking of temporal and spatial population changes, provided managers understand the value and limitations of the data (Burgess et al., 2016). Trained, expert-level participants in REEF’s Volunteer Fish Survey Project were able to expand the known range of 18 fish species and add *Gibbonsia metzi* to a previously established list of 260 fish species known to occur in the Salish Sea. Additionally, in the face of nearshore habitat loss, ocean acidification, warming ocean temperatures, shifting prey bases, and other threats to marine fishes, volunteer divers play an important role in helping state and federal agencies monitor species of concern, such as rockfishes (*Sebastes* spp.; Tolimieri et al., 2017). For example, two rockfishes documented on REEF surveys, *S. entomelas* — Widow Rockfish, and *S. nebulosus* — China Rockfish, are candidates for listing as species of special concern in Washington, and existing reports of *S. entomelas* within the Salish Sea are rare (Pietsch & Orr, 2019).

Based on our categorization system, 223 of 261 species known to occur in the Salish Sea are visually distinguishable, without the need for a specimen in hand. A large majority (102 of 120, or 85%) of the species most likely to be encountered and identified on a dive (category 1) were documented on REEF RDT surveys. An additional 36 species considered rare, uncommon in dive habitats, or visually similar to other species (category 2) were documented on RDT surveys. These records benefit from photographic evidence for verification, which is often provided to REEF during their quality assurance and control process. Many of the 81 undetected category 1 and category 2 fishes, while visually identifiable if encountered, were likely not documented by REEF volunteers because of the rarity of the species in the Salish Sea or because of a preferred habitat, depth range, behavior, or crypticity not well-suited for RDT. For example, the Pacific Viperfish (*Chauliodus macouni*), a category 2 species, is unmistakable, but it will likely never be seen by a REEF diver because it typically lives in thousands of meters of water. The common thresher shark (*Alopias vlupinus*) is a universally recognizable fish (category 1) that has been documented in the Salish Sea. The life history of this shark, however, would typically preclude it from the most common dive habitats. Similarly, category 2 flatfishes like the Petrale Sole (*Eopsetta jordani*) and Arrowtooth Flounder (*Atheresthes stomias*) are identifiable but are likely underrepresented in REEF data because only a small percentage of RDT surveys (7%) are conducted in soft-bottom habitats.

The categorization system described here relies on having an expert (such as a taxonomist) familiar enough with local fishes to determine if a species could be identified visually by trained individuals or must be identified in-hand. Categories can be used for quality control and assurance by identifying potentially erroneous sightings or to verify sightings prior to their entry into databases, as well as to gauge the detection likelihood of any given species on a visual survey. A similar scoring system might be useful in other ecosystems monitored by trained volunteers to improve data collection rigor or inform diver education.

REEF divers sighted 62% (138 of 223) of the visually detectable species occurring in the Salish Sea, suggesting that citizen science data collection is a highly valuable monitoring tool for the majority of species in the region. Furthermore, REEF data are provided continuously at no cost to the government agencies entrusted to manage this marine ecosystem. While we did not explore temporal trends in this study, REEF surveys are conducted year-round and consistently year after year, and data are publicly accessible soon after surveys are conducted. This makes citizen science an appropriate tool for tracking fish species diversity and distribution over time.

## Conclusions

Trained recreational divers provide an important data stream for documenting fish species occupancy, abundance, and distribution in the Salish Sea. In just over 20 years, expert-level REEF citizen scientists spent 11,918 h underwater conducting 13,274 surveys, generating data that expanded the known distribution of 18 species and identified a species not previously included in the list of recognized fishes for the ecosystem. Furthermore, by assigning detectability scores to RDT survey data, we show that expert surveyors collected reliable data on 53% of the ecosystem’s total known fish species. While the degree to which visual survey methods are useful for biodiversity monitoring varies by ecosystem, monitoring data gathered by citizen scientists can be a highly beneficial resource for management agencies to supplement fisheries-dependent and agency-sponsored, fisheries-independent monitoring.

## Supplementary information

Below is the link to the electronic supplementary material.

Supplementary file1 (PNG 2235 KB)

## Data Availability

The dataset used in this study is freely available in the Reef Environmental Education Foundation (REEF) repository, www.REEF.org.
