# Supplementary figures and images for: Documenting fishes in an inland sea with citizen scientist diver surveys: using taxonomic expertise to inform the observation potential of fish species
Source: Environ Monit Assess. 2022 Feb 26;194(3):227. doi: 10.1007/s10661-022-09857-1 (PMC8882091; doi:10.1007/s10661-022-09857-1)

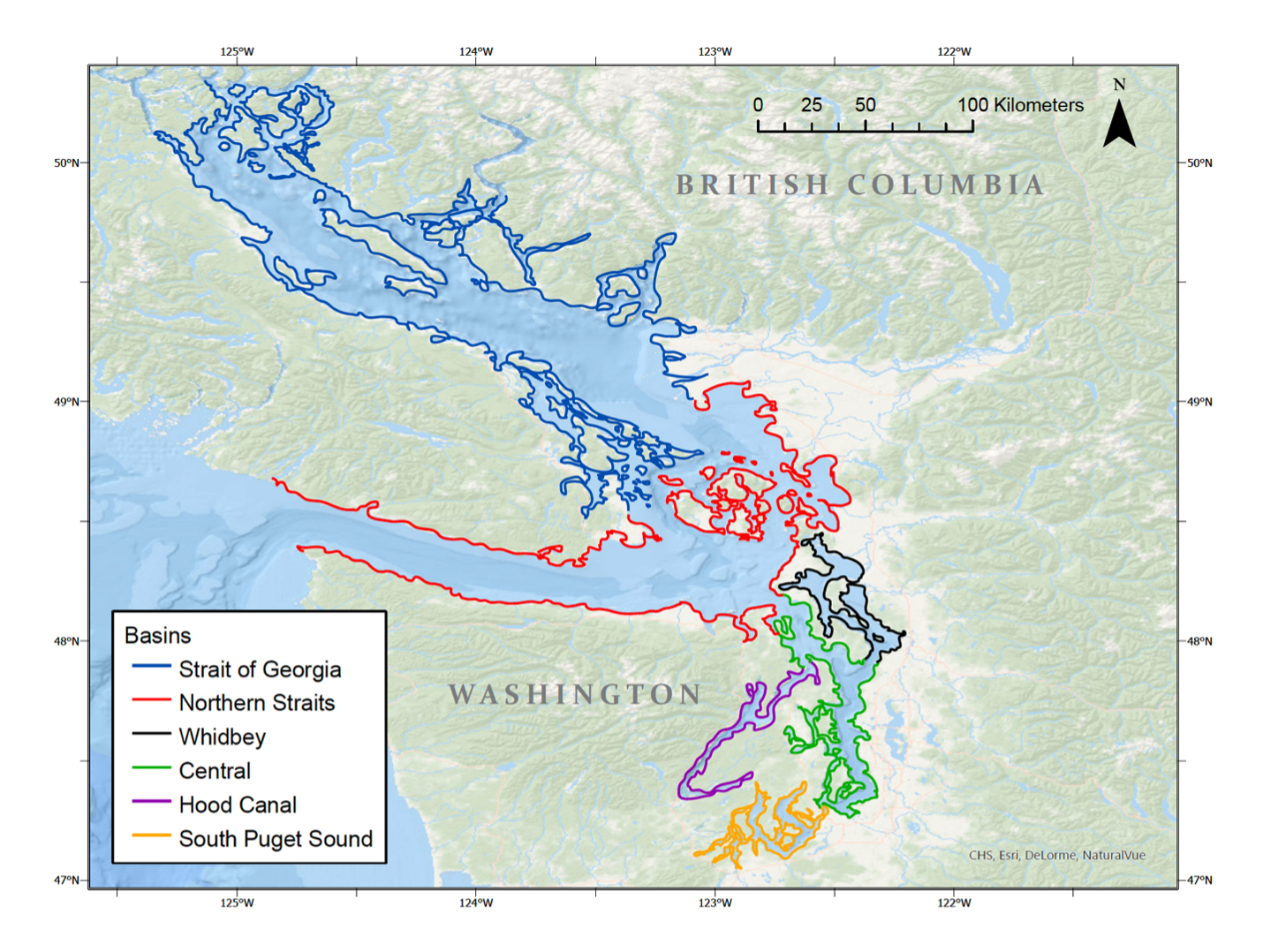

Supplement: Supplementary file 1 — Supplementary file1 (PNG 2235 KB) [file 10661_2022_9857_MOESM1_ESM.png]
